# Relationship between normalized distributional pattern and functional outcome in patients with acute cardiogenic cerebral embolism

**DOI:** 10.1371/journal.pone.0210709

**Published:** 2019-01-15

**Authors:** Masatoshi Takagaki, Manabu Kinoshita, Atsushi Kawaguchi, Akira Murasawa, Kazutami Nakao, Hajime Nakamura, Haruhiko Kishima

**Affiliations:** 1 Department of Neurosurgery, Kawachi General Hospital, Osaka, Japan; 2 Department of Neurosurgery, Osaka International Cancer Institute, Osaka, Japan; 3 Department of Neurosurgery, Osaka University Graduate School of Medicine, Osaka, Japan; 4 Center for Comprehensive Community Medicine, Faculty of Medicine, Saga University, Saga, Japan; McLean Hospital, UNITED STATES

## Abstract

This study aimed to elucidate spatial characteristics for magnetic resonance imaging (MRI) of cardiogenic cerebral embolism, to determine imaging biomarkers predicting patient outcome and cerebral herniation in cardioembolic stroke. This retrospective study assessed 90 patients with cardiogenic cerebral embolism. All images from MRI were normalized using a voxel-based symptom lesion mapping technique. Patients were categorized into two subgroups based on the outcome and presence of cerebral herniation. Each subgroup was assessed individually. The distribution map of all analyzed patients revealed accumulated ischemic lesions in bilateral middle cerebral artery areas. Ischemic lesions for the poor outcome group accumulated at the corona radiata on the right side and throughout the entire left hemisphere. Receiver operating characteristic (ROC) analysis suggested that a normalized ischemic volume of 62.8 mL allowed optimal differentiation between good and poor outcomes (sensitivity, 0.923; specificity, 0.923; area under the curve (AUC), 0.91) for left-side-dominant infarction. The distribution map for the cerebral herniation group revealed large ischemic areas in the left hemisphere. The analysis of differential involvement map with random permutation analysis showed that left anterior circulation infarcts were associated with midline shift. Receiver operating characteristic analysis revealed that a normalized infarction volume of 192.9 mL was highly predictive of cerebral herniation (sensitivity, 0.929; specificity, 0.750; AUC, 0.895). The medial frontal and occipital lobes, caudate head and basal ganglia were significantly involved in those patients who developed cerebral herniation. Ischemic volume contributed to outcomes and cerebral herniation. Ischemic lesions of the anterior and posterior cerebral arteries and basal ganglia in addition to middle cerebral artery area were identified as differences on MRI images between with and without cerebral herniation patients.

## Introduction

Recent advances in interventional devices have dramatically improved treatment outcomes in patients with acute cardiogenic cerebral embolism [1]. However, prediction of outcomes from imaging conducted early after ischemic stroke onset is imperative because a subset of patients over the golden time period < 180 min after the onset still suffer poor outcomes.

Voxel-based lesion-symptom mapping (VLSM) is a valuable technique to assess the relationship between locations of brain damage and symptoms [2]. In VLSM, results from individual magnetic resonance imaging (MRI) are warped and registered to a standard MRI brain atlas. As each individual brain is spatially normalized into common space, a cross-patient group analysis can be performed while ignoring patient-specific brain characteristics such as size and shape. In stroke imaging, lesion patterns of ischemia on diffusion-weighted imaging (DWI) represent a critical factor facilitating precise diagnosis of the ischemic stroke subtype [3]. Both stroke infarct location and volume after interventional recanalization therapy are reportedly closely associated with clinical outcomes using the VLSM technique [4–7]. Few studies, however, have reported frequency maps created using patients with cardiogenic embolic stroke, and this type of analysis remains scarce.

We hypothesized that the normalized ischemic volume, which can be measured only after normalization of individual brains to a standard brain atlas, would be predictive of patient outcome and cerebral herniation. This study aimed to determine the spatial distribution of DWI infarct lesions in patients with cardiogenic cerebral embolism via VLSM and attempted to determine imaging biomarkers predictive of patient outcome and cerebral herniation in cardioembolic stroke.

## Materials and methods

### Patient selection

The present study was approved by the institutional review board of Kawachi General Hospital. The requirement to obtain informed consent was waived. This retrospective study assessed 112 patients who underwent treatment for cardiogenic cerebral embolism between January 2011 and December 2014. We used the ASCO (atherothrombosis, small vessel disease, cardiac causes, and other cause) classification, which highly corroborates the TOAST classification, to select patients with cardiogenic cerebral embolism in the present study [8]. We enrolled patients with cardioembolic stroke grade C1 in the ASCO classification (107 cases), but excluded patients with atherothrombotic stroke grade A1 (3 case), small vessel stroke grade S1 (1 case), resulting in the accumulation of 103 patients. There were no cases with other causes grade O1. We then excluded 9 patients who were unable to undergo MRI because of pacemaker implantation. In the end, we examined 94 patients overall.

### Image analysis

MRI was performed with a 1.5-T system (Excelart Vantage Atlas; Toshiba, Japan) within 3 days after onset. Parameters for DWI sequences were: repetition time, 5100 ms; echo time, 95 ms; b-factor, 1000 s/mm^2^; slice thickness, 6.0 mm; gap, 1.2 mm. We normalized acquired DWI data from the 94 included patients using a method described earlier [9,10]. Digital Imaging and Communications in Medicine data from DWI images were converted to the Neuroimaging Informatics Technology Initiative (NIfTI) format using VINCI software (http://www.nf.mpg.de/vinci3/). An experienced neurosurgeon (M.T.) blinded to clinical data analyzed imaging studies and determined the voxels of interest in the ischemic area by manual lesion tracing on DWI using an in-house-developed software written in MATLAB R2015 (MathWorks, Natick, MA). DWI data were then registered to the MNI152 brain atlas provided by the Montreal Neurological Institute via a mutual information algorithm with a 12-degree-of-freedom transformation using the Functional Magnetic Resonance Imaging of the Brain (FMRIB) Software Library/FMRIB Linear Image Registration Tool (FSL-FLIRT; http://fsl.fmrib.ox.ac.uk/fsl/fslwiki/FSL). Visual confirmation after image registration was performed in all patients; some required rough manual adjustment before using FSL-FLIRT to obtain accurate registration. After completing the image registrations mentioned above, voxels of interests created beforehand were registered and resliced to MNI152 using the affine transformation matrix calculated for registering DWI to MNI152. Furthermore, all voxel of interests were summed and superimposed on the reference MNI152 to create a frequency map of the ischemic lesion, followed by measuring the volume of ischemic lesions registered to MNI152 as the normalized ischemic volume.

### Clinical assessment and subgroup analysis

We collected information regarding age, sex, stroke risk factors (e.g., hypertension, hyperlipidemia, diabetes mellitus, atrial fibrillation, and heart failure), clinical outcome using the modified Rankin scale (mRS), presence of cerebral herniation and pneumonia, and use of intravenous tissue plasminogen activator from all patients. Clinical outcomes were assessed either after 90 days or at discharge based on the mRS. Cerebral herniation was defined as presence of a significant midline shift. Patients were categorized into 2 subgroups based on the outcome and presence of cerebral herniation. Each subgroup was assessed individually in the present study.

### Statistical analysis

All statistical analyses were performed using EZR (Saitama Medical Center, Jichi Medical University, Saitama, Japan), a graphical user interface for R (The R Foundation for Statistical Computing, Vienna, Austria). Precisely, EZR is a modified version of R Commander designed to add statistical functions frequently used in biostatistics [11]. Categorical variables were analyzed using Fisher’s exact test; and ordinal variables with the Mann-Whitney test. We considered a threshold level of 0.05 as statistically significant for all analyses. In addition, ROC analysis was performed for the NIV, and the best operating point was determined by Youden’s index. Furthermore, multivariate logistic regression and backward elimination were performed, including all clinical characteristics and infarcted volume (S1 Table).

Analysis of differential involvement (ADIFFI) with random permutation analysis was used to reveal the statistical significance of differences in ischemic lesions between each subgroup, according to a previously described method [12,13]. Briefly, a voxel-wise 2-tailed Fisher’s exact test for a 2-by-2 contingency table, which compared each subgroup and lesion-positive with lesion-negative, was conducted within all voxels containing at least one lesion occurrence; the p value threshold was set at 0.05. A total of 500 cluster-based permutations were then performed. Results from random permutation analysis performed 500 times suggested that the chance of obtaining clusters larger than 114.9 mL in the outcome subgroup and 135.0 mL in the herniation subgroup were <5%. A cluster-size exceeding either of those threshold values was thus considered statistically significant.

## Results

The present study successfully normalized MRI-DWI images in 90 of the 94 patients. Failures of normalization were attributed to motion- or metal-induced artifacts. Table 1 summarizes patient characteristics. Mean age of the 90 patients enrolled in the present study was 75.9 years, and females constituted 55.4% of the cohort. The good outcome subgroup (mRS 0–2) comprised 32 patients, and the poor outcome subgroup (mRS 3–6) comprised 58 patients. Normalized ischemic volume was >145 mL in 36 patients; 13 of those 36 patients developing cerebral herniation, while the remaining 23 did not. Raw data are available in S1 Dataset.

**Table 1:** Patient demographic and treatment data.

		Outcome subgroups			Herniation subgroups		
	All patients (n = 90)	mRS 0–2 (n = 32)	mRS 3–6 (n = 58)	p value	Without herniation (n = 76)	With herniation (n = 14)	p value
Age (years)	75.9	71.4	78.4	**<0.001**	76.0	75.7	0.85
Female (%)	55.4	37.5	63.8	**0.026**	52.6	64.3	0.562
Pre mRS	0.6	0.2	0.8	**0.031**	0.7	0.1	0.053
Past history							
HT (%)	56.7	46.8	62.0	0.188	55.3	64.2	0.573
HL (%)	31.1	21.9	37.9	0.159	28.9	50.0	0.133
DM (%)	26.7	26.7	28.1	1.000	26.3	28.6	1.000
Af (%)	98.9	100	98.3	1.000	98.7	100	1.000
HF (%)	27.8	15.6	34.5	0.084	25.0	42.9	0.2
Onset to MRI (days)	1.3	1.1	1.4	0.246	1.5	0.3	**0.015**
Brain herniation (%)	15.6	0	24.1	**0.002**	-	-	
Pneumonia (%)	20	6.7	27.6	**0.025**	18.4	28.6	0.467
Major artery occlusion	47.8	12.5	67.2	**<0.001**	39.5	92.8	**<0.001**
Hemorrhagic transformation	4.4	0	6.9	0.293	0	28.6	**<0.001**
iv tPA (%)	27.8	18.8	32.8	0.22	22.4	57.1	**0.019**
Post mRS	3.3	1.2	4.4	**<0.001**	2.9	5.5	**<0.001**

**Abbreviations**: mRS, modified Rankin scale; HT, hypertension; HL, hyperlipidemia; DM, diabetes mellitus; Af, atrial fibrillation; HF, heart failure; iv tPA, intravenous tissue plasminogen activator. Pre mRS was evaluated before onset of stroke. Post mRS was evaluated either 90 days after stroke onset or at discharge.

### Voxel-based lesion-symptom mapping of cardioembolic stroke

**Fig 1:**
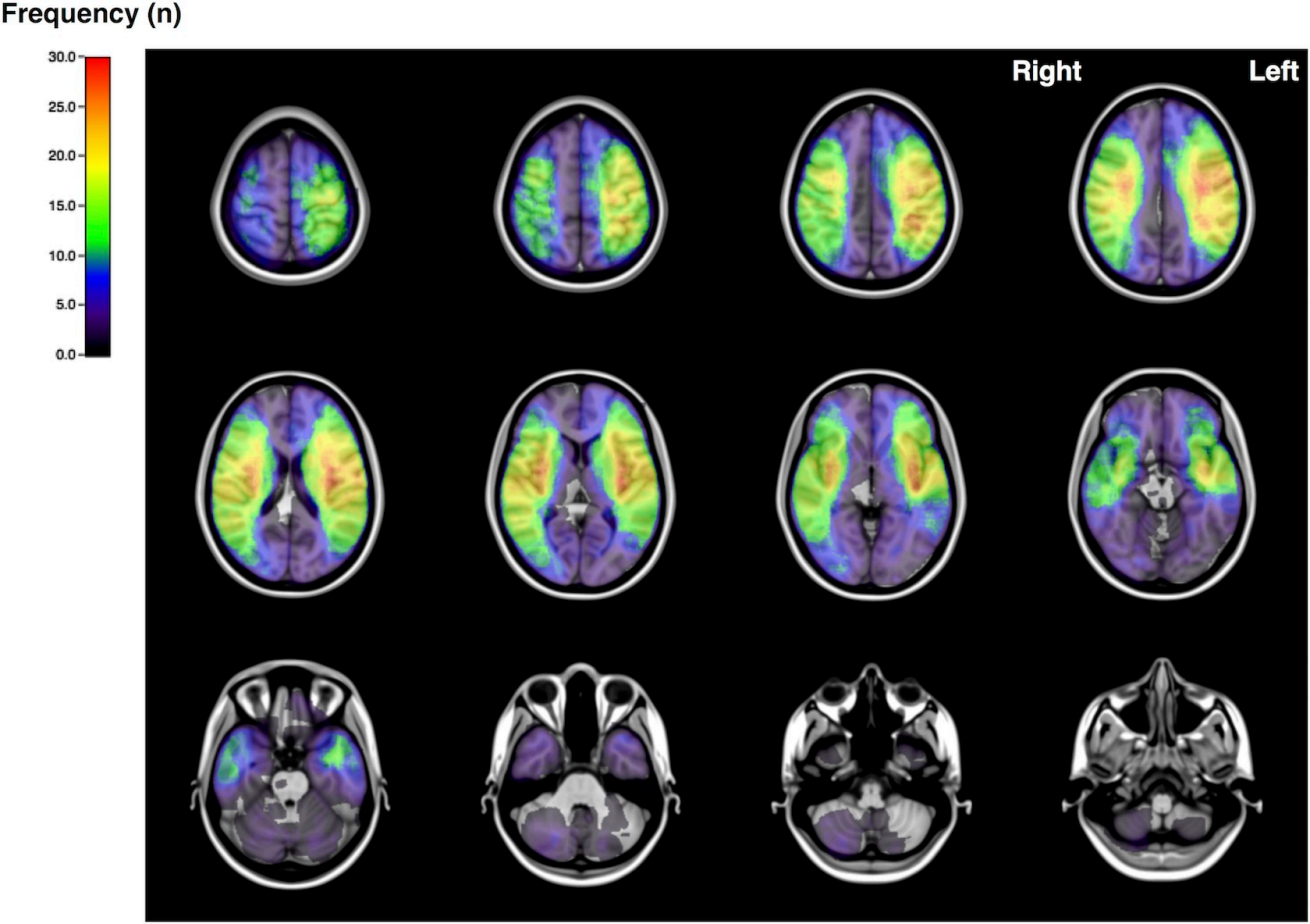
Axial Images of the frequency map for all cases. Note that lesions are mostly concentrated in MCA territories.

The distribution map of all analyzed patients revealed accumulated ischemic lesions in bilateral middle cerebral artery areas, especially the motor cortex and corona radiata (Fig 1). While 63 patients had ischemic lesions in the right cerebral hemisphere, 61 had those lesions on the left side, with 36 patients showing bilateral lesions. Mean ischemic volume after normalization was 103.9 mL on the right side and 131.9 mL on the left side. In the cerebellum, mean ischemic volume after normalization was 15.7 mL (7 patients) on the right side and 11.2 mL (7 patients) on the left side. We observed no significant difference in mean ischemic volume between right and left sides in either cerebral or cerebellar hemispheres (*P* > 0.05, Table 2).

**Table 2:** Infarction volume in each hemisphere.

	Right hemisphere	Left hemisphere	p value
Cerebral hemisphere			
average ± SD (ml)	103.9 ± 139.6 (n = 63)	131.9 ± 167.4 (n = 61)	0.363
Cerebellar hemisphere			
average± SD (ml)	15.7± 18.9 (n = 7)	11.2 ± 24.9 (n = 7)	0.128

### Correlation between outcome and ischemic lesion characteristics

**Fig 2:**
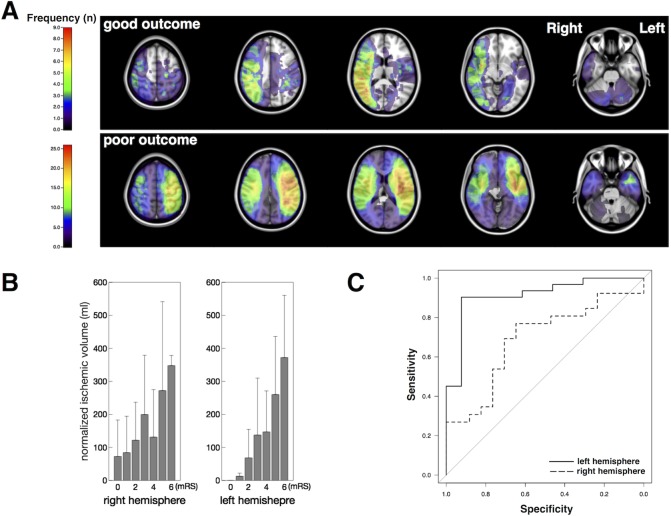
Subgroup analysis of outcome. **A)** Axial image of the frequency map of subgroups. Upper row, good outcome group; lower row, poor outcome group. Lesions in the good outcome group were concentrated strongly in the right hemisphere. **B)** Bar graphs depicting ischemic volumes in right and left hemisphere by outcome. **C)** ROC curves of ischemic volumes in the right and left hemispheres for predicting poor outcome (left side, 62.8 ml; sensitivity, 0.923; specificity, 0.903; AUC, 0.913; 95% confidence interval (CI), 0.817–1).

The distribution map for the good outcome group revealed accumulated ischemic lesions unilaterally in the right parietal lobe and right temporal lobe (Fig 2A, upper row). In contrast, ischemic lesions in the poor outcome group accumulated at the corona radiata on the right side and in the entire left hemisphere (Fig 2A, lower row). Normalized ischemic volume on the dominant infarction side correlated positively with mRS in both hemispheres (Fig 2B). ROC analysis revealed that normalized ischemic volume was more predictive of outcomes measured by mRS on the left than on the right (Fig 2C). The best operating point for normalized ischemic volume was 62.8 mL to effectively differentiate between good and poor outcomes (sensitivity, 0.923; specificity, 0.923; AUC, 0.91) for left-side-dominant infarction. The ADIFFI map with random permutation analysis showed no clusters exhibiting significant differences.

### Correlation between cerebral herniation and ischemic lesion characteristics

**Fig 3:**
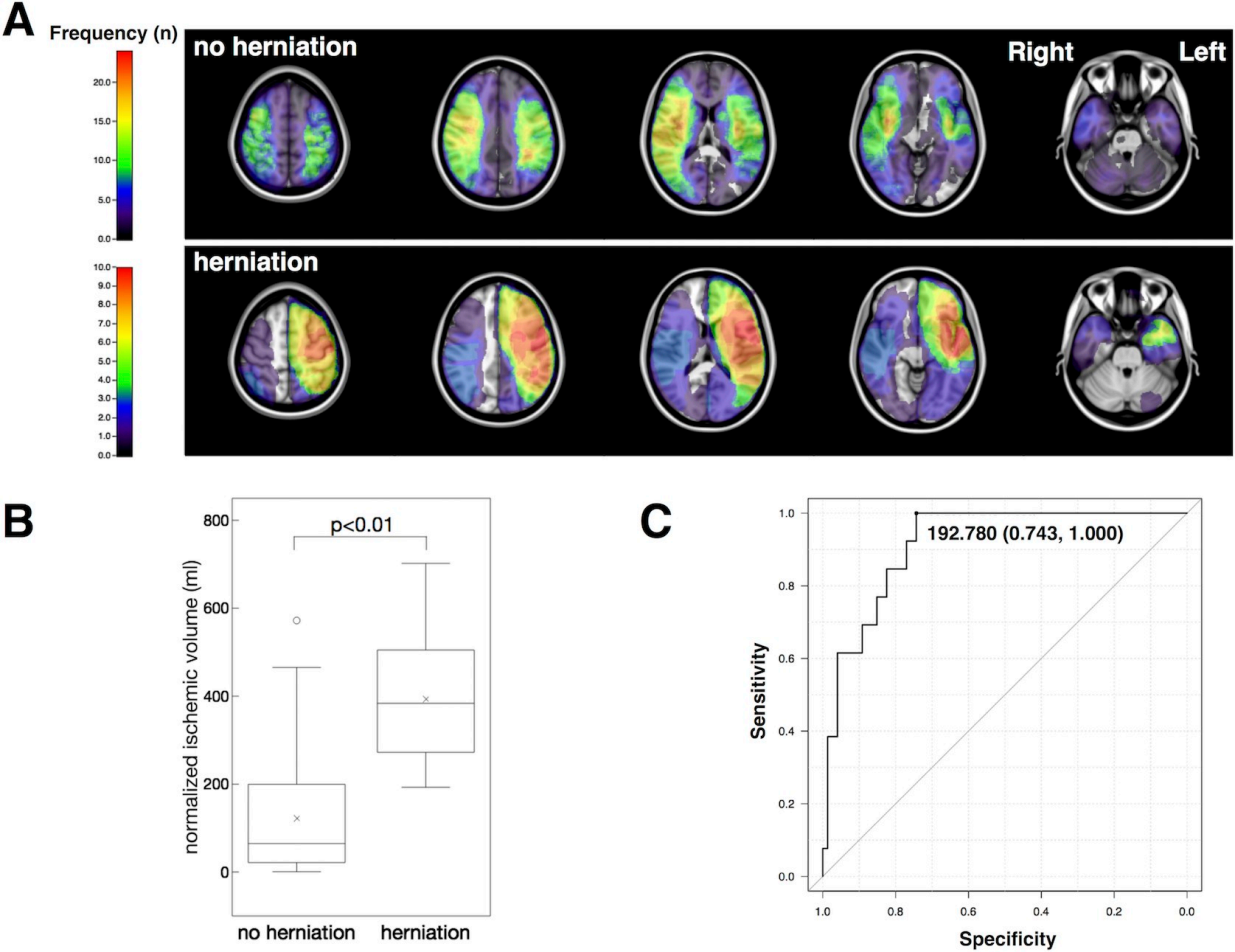
Subgroup analysis of cerebral herniation. **A)** Axial image of the frequency map of subgroups. Upper row, no-herniation group; lower row, herniation group. Lesions in the cerebral herniation group were larger and concentrated in the left hemisphere. **B)** Box-plot depicts ischemic volume of the dominant hemisphere with or without cerebral herniation. **C)** ROC curve for ischemic volume of the dominant hemisphere in predicting cerebral herniation. Threshold 192.8 ml: sensitivity, 1.000; specificity, 0.743; AUC, 0.916; 95%CI, 0.852–0.979.

**Fig 4:**
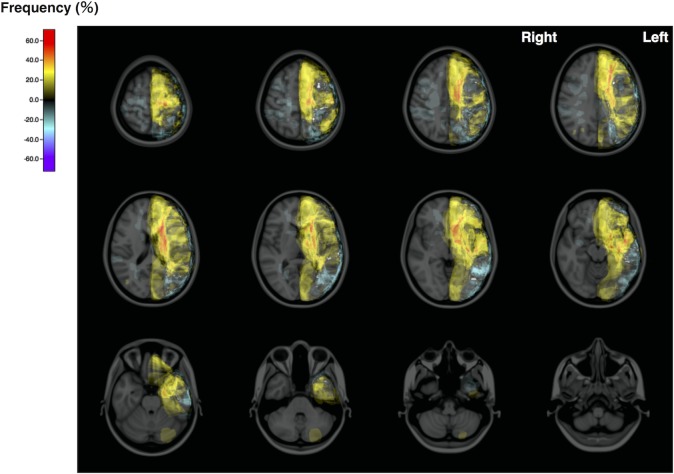
Spatial differences between cerebral herniation subgroups. Axial images of the frequency map of difference between with and without cerebral herniation groups (infarction volume >145 ml). All lesions are converted to the left side. ACA area and basal ganglia are clearly visualized as the difference. Color bar represents the difference in frequency expressed as a percentage.

**Fig 5:**
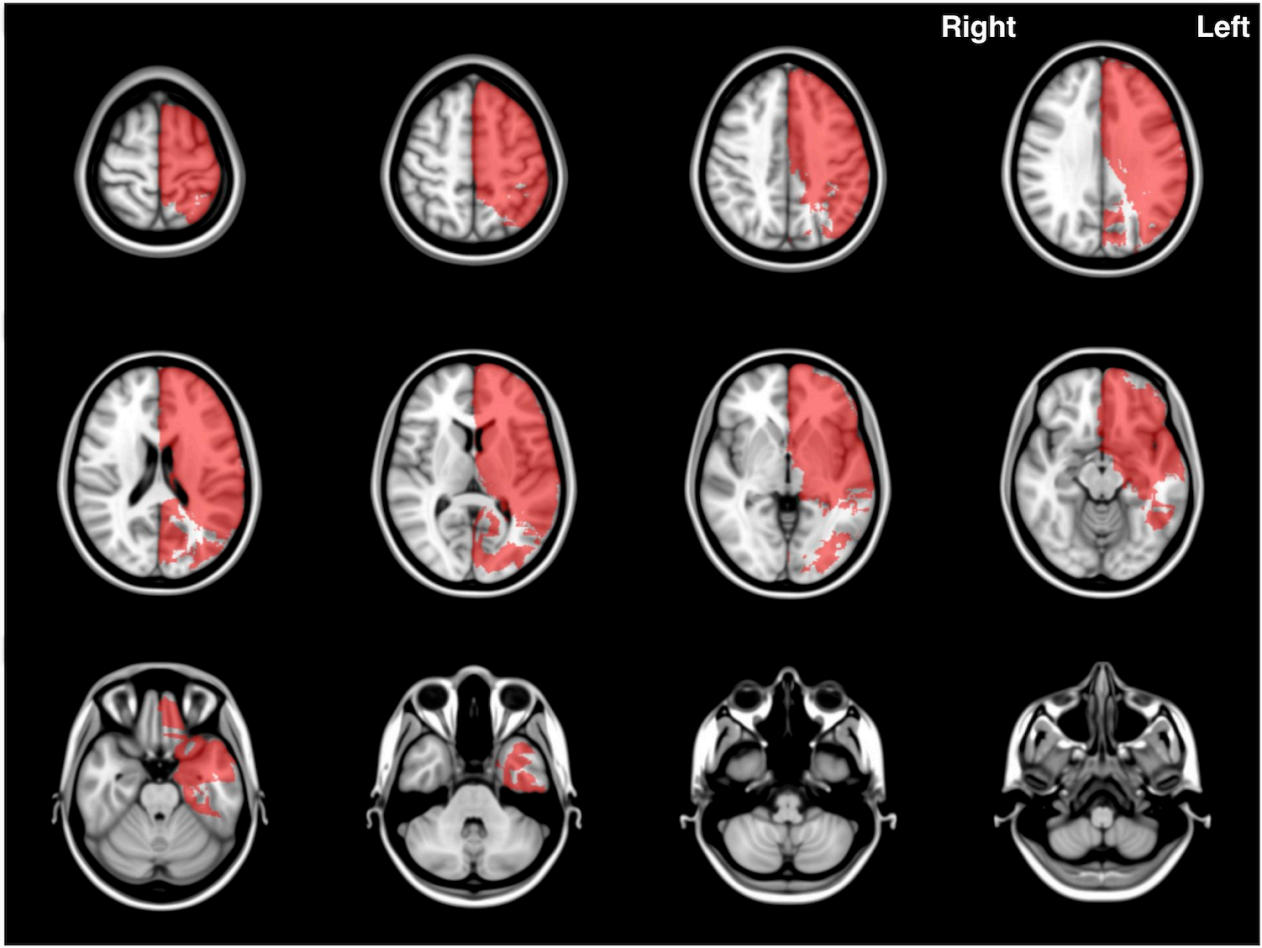
Analysis of differential involvement (ADIFFI) with random permutation analysis of herniation subgroup. Axial image of the ADIFFI map shows a large cluster in the left hemisphere that exhibited significant differences.

The distribution map for the cerebral herniation group revealed large ischemic areas in the left hemisphere (Fig 3A, lower row). Mean normalized ischemic volumes of the herniation and no-herniation groups were 410.0 and 315.0 mL, respectively. Normalized ischemic volume was significantly greater in the herniation group than in the no-herniation group (Fig 3B). ROC analysis revealed that a normalized ischemic volume >192.9 mL was highly predictive of cerebral herniation (sensitivity, 0.929; specificity, 0.750; AUC, 0.895; Fig 3C). Fig 4 illustrates spatial differences in ischemic areas between the herniation and no-herniation subgroups. Ischemic lesions with normalized ischemic volume >145 mL were all flipped to the left side in the present analysis. The medial frontal and occipital lobes, caudate head and basal ganglia were involved for those patients who developed cerebral herniation (Fig 4). The ADIFFI map with random permutation analysis showed a large cluster in the left hemisphere that exhibited significant differences between the herniation and no-herniation subgroups. (Fig 5).

## Discussion

This study aimed to elucidate spatial characteristics of DWI in patients with cardiogenic cerebral embolism using the VLSM technique and to investigate the characteristics of clinical subgroups. In the present study, DWI was successfully normalized in nearly all patients (90 of 94). Accordingly, we established a frequency map of ischemic lesions of cardiogenic cerebral embolism and revealed that these lesions were concentrated in middle cerebral artery areas, and that the ischemic volume of the left cerebral hemisphere was greater than that of the right hemisphere. In subgroup analysis, the lesion map of the good outcome group revealed a high frequency of ischemic lesions in the right parietal and temporal lobes. In addition, involvements of the anterior cerebral artery territory and basal ganglia were identified as differing between the herniation and no-herniation subgroups. Voxel-wise ADIFFI results basically showed that anterior circulation infarcts are associated with midline shift. Normalized ischemic volume was hypothesized to represent a good predictor of the outcome and cerebral herniation, as supported by ROC analysis, which revealed high sensitivity and specificity for good outcome and cerebral herniation.

### Propensity and ischemic volume

Several studies have reported the propensity for sidedness of cardiogenic cerebral embolism [14–17]. Whereas the left hemisphere propensity of cardiogenic emboli has been reported [17], the right hemisphere propensity of cardiogenic emboli has also been clinically and experimentally demonstrated [14,15]. In fact, a recent study has reported the predilections of cardiogenic embolic transport using a volumetric flow model [16], wherein researchers have proposed the cause of right propensity as the larger diameters of the brachiocephalic artery compared with the left carotid artery and linear divergence from the ascending aorta [15,16]. In addition, medium-sized (<1 mm) cardiogenic emboli reportedly move up the outer aspect of the arch, and the brachiocephalic artery as the first branch was supplied with the highest number of particles [16]. Contradicting these previous reports, no apparent propensity was observed in our results. Although the reasons for this discrepancy are unknown, small and medium-sized cardiogenic emboli in the non-dominant right hemisphere failed to cause symptomatic cerebral infarction, which could have biased patient selection [14]. In the present study, normalized ischemic volume was larger in the left hemisphere than in the right hemisphere. Carr et al. reported that relatively larger emboli are more likely to prefer the descending aorta based on volumetric flow analysis [16], indicating that larger emboli tend to travel along the “left” common carotid artery branching from the aorta distal to the brachiocephalic artery, which can cause a larger infarction volume in the left hemisphere.

### Distribution and outcomes

Lesion maps of the good and poor outcome groups differed in the present study. Stroke lesions in the right hemisphere, which is usually the non-dominant side, with corresponding symptoms of spatial-constructive disorders, neglect, anosognosia, and agnosia scores, resulted in a much lower National Institutes of Health Stroke Scale score than those in the left hemisphere as the language-dominant side; this difference in neurological severity has been associated with the outcome as predicted [18]. Ischemic locations in the good outcome group were concentrated in the right hemisphere, particularly the parietal and temporal lobes; this distribution map on normalized brain images from MRI indicated that the non-eloquent area correlates with good outcome in patients with cardioembolic stroke. Cheng et al. reported that infarct lesions involving the corona radiata, internal capsule, and insula are closely associated with poor outcome in patients with stroke [6]. Similarly, the lesion map of the poor outcome group revealed a high frequency of ischemic lesions involving the corona radiata. In addition, ischemic lesions with a substantial impact on the left hemisphere involve the speech area, suggesting that ischemic lesions related to motor and speech function contribute to patient outcome. On the other hand, several studies have reported the relationship between ischemic volume and outcome [18–21]. Yoo et al. reported that a final infarct volume of approximately 50 mL after intra-arterial therapy using interventional devices offers a good threshold to distinguish good from poor outcome [20]. Likewise, Zaidi et al. reported that final infarct volume after endovascular therapy in patients with MCA occlusion differentiates good and poor outcomes [21]. Based on these findings, the final infarct volume cutoff between good and poor outcomes could plausibly be around 40–50 mL [21]. Conversely, several studies have also reported regarding the prediction of outcomes from the initial infarct volume. Albers et al. reported that a pretreatment ischemic volume of DWI >100 mL predicts a poor clinical outcome [22]. A study on acute ischemic stroke with recanalization therapy revealed that a pretreatment DWI volume >70 mL indicated poor clinical outcomes [23]. However, mechanical thrombectomy for major intracranial artery occlusion, exerts a significant impact on outcomes. Precise prediction of patient outcomes from the pretreatment ischemic volume is challenging [21]. In the present study, no patients were treated by mechanical thrombectomy, which ensured a uniform patient cohort. Ischemic volume after normalization of >62.8 mL of the left hemisphere was demonstrated to predict poor outcome with high sensitivity and specificity.

### Cerebral herniation

Previous research has mentioned that malignant middle cerebral artery occlusion inducing cerebral herniation with external decompression has a right propensity, but ischemic etiology has not been mentioned [24,25]. On the other hand, in the present study, the ischemic area in the cerebral herniation subgroup was concentrated in the left hemisphere and was large, including the anterior cerebral artery area, in patients with cardioembolic stroke. As mentioned earlier, mean ischemic volume was greater in the left hemisphere than in the right, which might have contributed to the different propensity compared with previous reports. Cerebral herniation signifies large ischemic stroke involving the middle cerebral artery area [26–28]. Many studies have reported defining 145 mL of acute ischemic volume as the cutoff value for malignant middle cerebral artery occlusion [25,27,28]. Oppenheim et al. reported that an ischemic volume >145 mL in patients with middle cerebral artery or carotid T occlusion is the best predictor, which attains 100% sensitivity and 94% specificity for malignant infarction. Furthermore, Goto et al. reported the ischemic volume threshold predicting malignant middle cerebral artery infarction in patients >75 years old patients as 102 mL [29]. In addition, they noted that age-related brain atrophy makes a difference to the threshold. Hence, they used the ratio between the ischemic volume and individual brain volume. Although the present study did not evaluate age-related brain atrophy, ischemic volume in each patient was normalized using VLSM. The present study demonstrated normalized ischemic volume in patients with cardioembolic stroke as a novel predictor of cerebral herniation, with a cutoff value of 192.9 mL for optimal differentiation. In addition, we successfully elucidated the difference in distribution maps between herniation and no-herniation subgroups. Consequently, the anterior cerebral artery area was revealed as the difference; likewise, the caudate head and basal ganglia were clearly visualized [30].

## Conclusions

We normalized data from DWI of patients with acute cardiogenic embolic stroke and demonstrated a relationship between ischemic lesions characteristics and clinical features. Normalized ischemic volume was greater in the left hemisphere than in the right hemisphere and contributed to poor outcome. Moreover, ischemic lesions of the anterior and posterior cerebral artery and basal ganglia in addition to middle cerebral artery area were identified as differences on MRI images between with and without cerebral herniation patients.

## Supporting information

S1 Table Multivariate logistic regression analysis. (DOCX)

S1 Dataset Raw data of patient characteristics and normalized ischemic volume. (ZIP)
