# Supplementary material for: Relationship between normalized distributional pattern and functional outcome in patients with acute cardiogenic cerebral embolism
Source: PLoS One. 2019 Jan 15;14(1):e0210709. doi: 10.1371/journal.pone.0210709 (PMC6333406; doi:10.1371/journal.pone.0210709)
Supplement: S1 Table — (DOCX) [file pone.0210709.s001.docx]

|  | Odds ratio | 95%CI | p value |
| --- | --- | --- | --- |
| Herniation subgroup |  |  |  |
| infarction volume | 1.010 | 1.010-1.020 | <0.001 |
| heart failure | 6.870 | 1.110-42.4 | 0.038 |
| Outcome subgroup |  |  |  |
| age | 1.140 | 1.060-1.220 | <0.001 |
| infarction volume | 1.010 | 1.010-1.020 | <0.001 |
